# A Multimodal Framework for Understanding Perceptual Segmentation of Natural Scenes In Autism

**DOI:** 10.64898/2025.12.17.695033

**Published:** 2025-12-18

**Authors:** Darnell K. Adrian Williams, Tridib K. Biswas, Concetta Brusco, Sophie Molholm, Ruben Coen-Cagli

**Affiliations:** 1. Dominick P. Purpura Department of Neuroscience, Albert Einstein College of Medicine, Bronx, NY, USA; 2. Department of Psychiatry and Behavioral Sciences, Albert Einstein College of Medicine, Bronx, NY, USA; 3. Department of Pediatrics, Albert Einstein College of Medicine, Bronx, NY, USA; 4. Department of Systems & Computational Biology, Albert Einstein College of Medicine, Bronx, NY, USA; 5. Department of Ophthalmology & Visual Sciences, Albert Einstein College of Medicine, Bronx, NY, USA

**Keywords:** Autism, Visual Segmentation, EEG, Eye-Tracking, Natural Scenes, Perceptual Dynamics

## Abstract

Understanding how individuals segment complex natural scenes into perceptual segments is essential for explaining both typical and atypical sensory processing. In Autism Spectrum Disorder (ASD), differences in visual segmentation and integration are widely documented. However, existing paradigms often rely on simplified stimuli and do not capture the dynamic, naturalistic processes underlying real world scene segmentation. To overcome those limitations, in this work we present a novel, multimodal experimental framework. The framework combines precisely timed, trial-based perceptual measurements with electroencephalography (EEG) to examine how neural activity aligns temporally with segmentation decisions and with eyetracking to examine visual exploration strategies and attention allocation. Neurotypical (NT) participants and participants with ASD aged 16 years and above viewed natural scenes and textures and indicated whether two cued image regions belonged to the same or different perceptual segments. Responses to multiple pairs of cues enabled the estimation of subjectively perceived segmentation maps and the associated uncertainty. Across NT and ASD populations, we analyzed segmentation maps, reaction time distributions, gaze profiles, and trial-aligned neural responses. Behavioral results revealed slower and more idiosyncratic segmentation patterns in ASD. Eye-tracking analyses also demonstrated distinct gaze patterns suggesting broader exploration of the images during early viewing. EEG analyses demonstrated delayed and more diffuse occipital activation in ASD, accompanied by reduced global field power at several key time windows spanning stimulus presentation. Together, these findings establish a reproducible method for studying the temporal and spatial components of natural scene segmentation and indicate meaningful behavioral and neural distinctions in ASD. This framework provides a methodological bridge between controlled experimental stimuli and realworld perception in both neurotypical and neurodivergent populations.

## INTRODUCTION

### Terminology note:

There is ongoing discussion within the autism community regarding whether person-first language (“individuals with autism”) or identity-first language (“autistic individuals”) is preferred. Current preferences vary across autistic adults, self-advocates, clinicians, and researchers. In this paper, we tend to use person-first language, consistent with conventions in clinical and neuroscience research, while recognizing and respecting that many in the autism community prefer identity-first language. Our choice is not intended to imply a value judgment, and we acknowledge the validity of both approaches.

Autism Spectrum Disorder (ASD) is a highly diverse, heritable, lifelong neurodevelopmental condition defined clinically by two core diagnostic criteria: (1) persistent impairments in social communication and interaction, and (2) restricted repetitive patterns of behavior^1,2^. Although not part of the diagnostic criteria, many individuals with ASD also show numerous differences in sensory perception, which are the focus of the present study^1–6^.

These sensory features typically present throughout development, are prevalent in individuals with autism regardless of intellectual ability, and vary widely. These atypicalities include both hyper- and hypo-sensitivities to the world around them^6,7^ and include a range of symptoms, such as heightened sensitivity to sound or light and, in some cases, remarkable perceptual talents^8–10^. Individuals with autism are often less susceptible to visual illusions^11^, may possess superior pitch memory compared to the general population^12^, and frequently demonstrate exceptional and unique performance in visual search and discrimination tasks^4,6,13–17^.

These sensory and perceptual differences can meaningfully affect the daily life of people with autism. Theories such as the Weak Central Coherence^13^ and the Enhanced Perceptual Functioning^6^ proposed that individuals with ASD tend to integrate visual information differently than neurotypical individuals^14,18,19^. This refers both to integration of information across different parts of the visual field or between different visual features, as well as more broadly to integrating bottom-up, sensory-driven signals and top-down signals that convey prior expectations^20^. These classical theoretical frameworks have contributed to the interest in sensory symptoms, driven in part by the possibility that the core social features of autism may stem from more fundamental differences in sensation and perception^21,22^. As a result, there is strong motivation to understand the neurobiology and mechanisms underlying these sensory and perceptual atypicalities, ranging from interactions between brain areas^17,23–26^ to interactions between neurons in recurrent circuits^27–29^.

In this article, we consider a widely used paradigm^30^ for studying visual integration, namely visual segmentation, which refers to the ability to distinguish objects from their background and from one another, and to interpret complex visual scenes^31,32^. This process is fundamental to perception and is shaped by both bottom-up sensory processing and top-down modulation^17,33–38^. Prior work has shown that individuals with ASD exhibit atypical patterns of visual segmentation, including slower facial and texture segmentation, which may contribute to broader differences in visual processing^4,6,14–17^. However, it remains unclear whether these effects extend to natural scenes, as most prior work has relied on highly simplified textures or coarse low-level stimuli. Examining segmentation in natural scenes is essential because it more accurately reflects the demands of everyday visual processing. This added complexity is thought to contribute to and shape some of the social and sensory differences often observed in ASD^21^. The first contribution of this article is to demonstrate that a recently published experimental paradigm^30^ that was validated in neurotypical (NT) participants, enables a precise characterization of natural image segmentation in ASD.

Our second contribution is to adapt the experimental paradigm to include simultaneous measurements of eye movements and brain activity. Individuals with ASD frequently display irregular eye movements and altered fixation patterns for socially and non-social visual stimuli^39–43^. This suggests that differences in how visual information is sampled, prioritized, and integrated may relate to perceptual differences. In addition, because perception and eye movements unfold rapidly and interactively, the ability to track neural activity in real-time is essential for understanding how sensory input is processed and integrated with attention and prior expectations^44–46^. This is particularly important in autism, where differences in sensory processing and perceptual integration are thought to arise from altered coordination between sensory-driven and higher-order brain activity^47–49^. For this reason, the experimental design presented here also includes electroencephalography (EEG). EEG captures brain activity with millisecond precision, allowing us to examine the temporal dynamics of perception as they occur.

**Figure 1.**
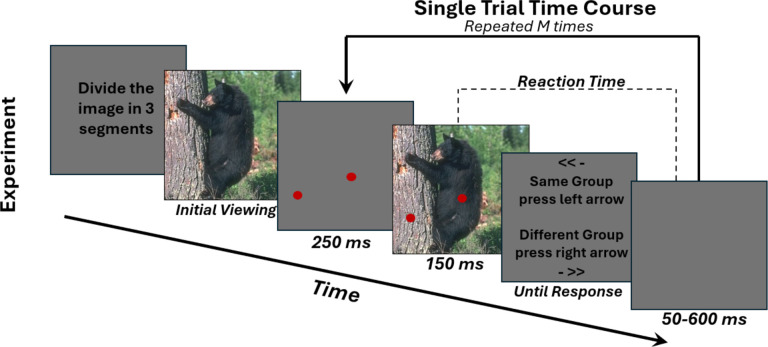
One experimental block of the segmentation task. Each block began with a text prompt instructing participants to mentally divide the scene into a predetermined number of segments. The image to be segmented was then displayed once and the participants viewed the scene freely. Next, a sequence of trials started. In each trial, two red circles were presented for 250 ms at pseudorandom locations on a grey screen, followed by the same image for 150 ms with the circles superimposed. Immediately afterward, a text prompt asked participants to indicate whether the parts of the image cued by the two circles belonged to the same segment or to different segments, using a left or right key press. Key mappings for “same” and “different” were randomized across participants but remained constant for each participant across trials and blocks. Reaction time was defined as the time elapsed between the offset of the image and the key press (participants were not allowed to respond before image offset). After the response, a grey screen was presented during an intertrial interval lasting between 50 and 600 ms.

**Figure 2:**
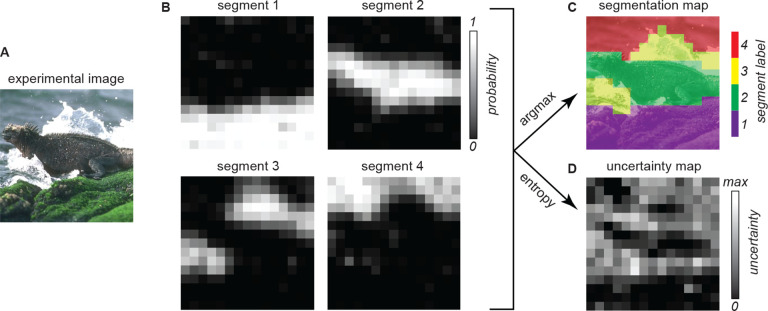
Example segmentation maps estimated from one experimental block. **(A)** The image used in this experimental block. **(B)** The probabilistic segmentation map estimated from the data of one participant. There is one map for each segment; within each map, the brightness of each pixel represents the probability that the pixel was assigned to the corresponding segment. **(C)** The segmentation map estimated by assigning each pixel to the segment with highest probability (i.e. using the argmax operator). Colors reflect segment identity, but not ordinal or semantic information. **(D)** The perceptual segmentation uncertainty map. Brightness corresponds to the entropy of the probability distribution over segment labels, which is a measure of perceptual uncertainty (brighter pixels denote higher uncertainty; the maximum possible value depends on the number of segments, K, and it is given by log(K)).

We employ a perceptual segmentation task in which, on each trial, participants are briefly presented with an image and they judge if a pair of cued locations on the image belongs to the same segment or different segments (Fig. 1). Data collected for several pairs of locations on one image enables the reconstruction of segmentation maps for each participant (Fig. 2). We integrate these measurements with recordings of gaze behavior and EEG-recorded neural activity. Unlike prior work on segmentation in ASD, we apply this paradigm to natural images as well as to synthetic textures. This multimodal framework provides high-resolution temporal and spatial data to compare perceptual organization, attentional allocation, and brain-wide dynamics across individuals and stimuli.

We demonstrate this paradigm with data collected from 24 NT and 13 ASD participants. We find that individuals with ASD are able to perform the segmentation task as well as NT participants, and their segmentation maps appear qualitatively comparable across both natural scenes and textures. This opens the door to the possibility that differences in the perception of segments may not be entirely reflected in the final outcome but rather in the processes and timing that lead to it. Consistent with this possibility, we find systematic differences in the timing and consistency (within and across individuals) of perceptual reports; in the strategies adopted for visual exploration, revealed by the spatial maps of eye movements; and in the dynamics and spatial distribution of visually evoked neural activity. These findings represent a useful entry point to investigate differences in perceptual segmentation in autism.

## METHODS

### Participants

We enrolled 24 typically developing (NT) participants and 13 participants with an autism spectrum disorder diagnosis (ASD). The NT cohort ranged in age from 16.3 to 29.7 years (mean 22.0 土 4.9) and included eleven female participants. The ASD cohort ranged in age from 16.4 to 31.1 years (mean 23.0 土 4.4) and included two female participants. All participants provided online informed consent prior to participation. All participants were native English speakers with normal or corrected-to-normal vision. A trained clinical psychologist confirmed ASD diagnoses.

The Institutional Review Board (IRB) of the Albert Einstein College of Medicine approved all study procedures. Participants were compensated according to institutional guidelines for their time in the laboratory. All procedures conformed to the ethical standards of the Declaration of Helsinki.

### Preregistration

This study was not preregistered.

### Behavioral data acquisition, preprocessing and analysis

#### Task description:

Figure 1 illustrates schematically one block of the experiment (detailed in Vacher et al., 2023^30^). The structure of each block was as follows. At the start of the block, participants were presented with on-screen text explaining the task instructions, namely to segment each image into K perceptual segments (K ranging from two to five, across blocks), and to provide responses using the left and right arrow keys.

After the text with the instructions, the image to be segmented was displayed allowing participants to freely view the image and decide how to segment it. Next, a series of short trials started. In each trial, participants were shown a pair of red circles on a gray screen of average luminance for 250 ms, followed by the same image with the circles overlaid for 150 ms. Both the image and the circles then disappeared, and a response screen appeared, prompting participants to indicate whether the two dots belonged to the same segment or different segments using a left or right key press. The key mappings for “same” and “different” were randomized between participants but remained identical in all the blocks performed by each individual participant. The response screen remained visible until a response was made. The time elapsed between the offset of the image and the key press was recorded as the reaction time, noting that participants were not allowed to respond before the offset of the image. After the response, a gray screen was presented during an inter-trial interval of random duration between 50 ms and 600 ms milliseconds, to minimize anticipatory responses (note that this interval includes also the time elapsed between key press and key release, which varied across trials and participants).

Before starting the experiment, the task was described verbally. Participants were told that there is no right or wrong segmentation, and that during the experiment they should base their answers on the segments they perceive. This was further illustrated by showing on a computer screen two example images and several possible segmentation maps of those images. Next, participants performed 25–50 practice trials to become familiar with the task structure. After confirming that they understood the task, participants performed up to 6 blocks in a single visit, with a unique image in each block (two participants from the ASD cohort completed two visits for a total of 12 blocks). Participants were offered short breaks every 3 minutes during a block and longer breaks between blocks. At the end of the experiment, participants were asked to describe verbally how they segmented the image. This information was recorded for each image.

The duration of a single block depended on the number of trials performed. This was set to the minimum number of trials needed to reconstruct a segmentation map with spatial resolution of 15×15 for the given number of segments K (using the equations derived in (30)). For K=2,3,4,5 participants performed 225, 450, 675, and 900 trials, respectively. The time to complete a block, including voluntary breaks, ranged from approximately 5 to 30 minutes.

#### Visual stimuli and presentation:

Visual stimuli were presented on a monitor (Dell UltraSharp 1704FPT) that was gamma calibrated and placed at a distance of either 67 cm or 96 cm. The images were rescaled to cover an area of 8.7×8.7 degrees of visual field. The viewing distance was controlled by using a chin rest. Stimulus presentation was controlled with custom Matlab code that used the PsychToolbox 3 toolbox^50,51^.

Each participant completed up to four blocks with natural images and up to two blocks with synthetic, parametric textures in a single visit. Natural images were selected from the Berkeley Segmentation Dataset 500^52^ (BSD500), a dataset of 500 natural scene images used in computer vision segmentation research. Images were pre-screened based on human-drawn segmentations provided with the BSD500 dataset, to exclude images with more than 10 distinct, hand-drawn segments. Next, 112 images were selected manually and cropped to a size 256×256 pixels. These 112 were divided into four categories, corresponding to the number of segments K requested at the start of the block. In this paper, we report data collected for five images from each category (i.e. 20 images total) with up to five NT and five ASD participants per image.

For the blocks involving artificial texture stimuli, we generated the images by first partitioning the image into two or three regions that were randomly shaped but spatially compact. We then filled each region with a distinct but similarly oriented texture. Textures were bandpass Gaussian noise textures, created as described in Vacher et al^30^. Each texture was synthesized by convolving a white Gaussian noise image with a spatially dependent filter, which produced the desired spectral properties in each region and resulted in oriented textures with controlled differences in spatial frequency and orientation content.

#### Estimation of segmentation maps and perceptual uncertainty:

The trial by trial responses collected during one experimental block were used to estimate the segmentation map of each participant and image. This was done using the method described in Vacher et al^30^. Briefly, the goal is to estimate the probability that each pixel in the image belongs to any segment. The number of segments is set to the same value that participants were instructed to use at the start of the block. Each segment is given an integer label, and the method uses numerical optimization to find the probabilistic assignments (termed probabilistic segmentation maps; Fig. 2B) that are most compatible with the responses of the participants. Next, the segmentation map is obtained by assigning each pixel to the segment label with the highest probability (Fig. 2C). Pixels with a probability close to 1 for one segment, and close to zero for all other segments, denote high perceptual certainty, i.e. the participant was highly certain in the assignment of that pixel. Conversely, pixels with a similar probability for all segment labels denote high perceptual uncertainty. This perceptual uncertainty was further summarized in the perceptual segmentation entropy map (Fig. 2D), which reports the entropy of the probability distribution over segment labels at each pixel. The estimation of the probabilistic maps was obtained using the Python implementation of software package Vseg^30^ with hyperparameter λ (which controls spatial smoothing of the map) set to 5.

#### Quantifying the similarity between maps:

We defined an Information Gain (IG) measure of similarity between two segmentation maps, as follows. For each image, the mutual information between two segmentation maps (i.e. the maps from two distinct participants) was calculated:

$$MI\left(\text{map}1;\text{map}2\right)=\frac{\sum_{m_{1}=1}^{M_{1}}\sum_{m_{2}=1}^{M_{2}}P\left(m_{1},m_{2}\right){\text{log}}_{2}\left(\frac{P\left(m_{1},m_{2}\right)}{P\left(m_{1}\right)P\left(m_{2},\right)}\right)}{\sqrt{H\left(\text{map}1\right)H\left(\text{map}2\right)}}$$

where *m*_1_ is the segment label in the first map (i.e. *m*_1_ ranges from 1 to the total number of segments *M*_1_in the first map) and similarly for *m*_2_; and *H* is the entropy of the map. The *MI* measures the similarity between maps because it quantifies how much the values of one map are predictable from the second map. This value was then compared to a null distribution of 10,000 values of *MI* between a randomly shuffled map (e.g. map 1) and the original version of the other map. This was done to construct a null distribution to determine significance of the *MI* value (one-tailed non-parametric permutation test, alpha=0.05). The shuffled maps were generated by randomly shuffling the spatial locations of map values, such that the marginal distribution of map values remained the same. Therefore, we quantified the similarity between maps as the percent information gained from the map spatial structure relative to the null distribution. The same procedure was used to compute similarity between two perceptual uncertainty maps.

#### Exclusion criteria:

In a small subset of experimental blocks (4/143 [2.8%] blocks for NT; 9/76 [11.8%] for ASD), the segmentation maps indicated that the participant had not performed the task correctly: data from those blocks was discarded. This was clearly indicated by segmentation maps that lack any spatial structure (e.g. Supp. Fig. 1A). Lack of spatial structure reflects that the responses were essentially random, i.e. unrelated to the position of the cues. We further confirmed this by estimating the segmentation maps from surrogate data where we randomly shuffled the measured responses relative to the cue pair presented in each trial: we observed that the segmentation map estimated from this surrogate data lacks spatial organization (e.g. Supp. Fig. 1B). Data from one additional block was excluded: after completing the block, the participant explained that they had partitioned the image into four quadrants rather than the segments they perceived (this was also evident from the estimated segmentation map; Supp. Fig. 1A).

#### Analyzing reaction times across blocks and participants:

Before analyzing reaction times, we removed trials in which responses were above the 90th percentile in the distribution of logtransformed RTs. We used median RTs as a measure of central tendency given that distributions were skewed. However, we found similar results using the arithmetic mean and the geometric mean. Confidence intervals around the median were found by bootstrapping with 9,999 resamples.

To perform the analysis of RT vs. trial number, we first z-scored the data. In other words, the mean RT for each unique case of a participant seeing a stimulus was set to 0, and the standard deviation to 1, to eliminate stimulus-specific or presentation-specific confounds.

In Fig. 6, to compute the number of trials required for a participant to halve their RT (which we term the trial constant) we fit the parameters *α* and *τ* in the following equation:

$$\text{RT}=\alpha \cdot 2^{-t/\tau }$$

Where RT is the reaction time, *α* is a scaling factor, *t* is the trial index, and *τ* is the positive-value trial constant. Parameters *α* and *τ* were fit using a nonlinear least-squares regression.

### Electroencephalography data acquisition and preprocessing

#### EEG Acquisition:

For a subset of participants (11 in the NT cohort and 9 in the ASD cohort), we monitored neural activity with scalp EEG recordings. Participants were seated in a dark, electromagnetically shielded booth (Industrial Acoustics Company Inc., Bronx, NY). Neural activity was recorded using a 64-channel BioSemi ActiveTwo EEG system (BioSemi, Amsterdam, The Netherlands). The EEG signal was continuously sampled at 512 Hz, and analog TTL triggers marking the beginning of the trials and the participant responses were transmitted from the stimulus computer to the acquisition system and logged in a dedicated channel within the EEG recording (we term this the event channel). This information was used to define epochs and align EEG data temporally to task events and gaze data.

#### EEG Pre-processing and Bad Channel Handling:

Raw data files in (.bdf) format were loaded into MNE-Python for pre-processing^53^. The event channel was preserved for event detection, and a standard BioSemi 64 montage was applied to assign 3D channel positions.

First, bad channels were detected using the PyPREP pipeline^54^, which employs a RANSAC-based detection algorithm to flag channels with high variance, flat signals, and poor correlations with neighboring channels. Visual inspection of channel variance plots was conducted for quality control. Identified bad channels were removed and subsequently interpolated from surrounding electrodes. Across files with available preprocessing metadata, the NT cohort had a mean of 2.00 bad channels per file (range = 0–3), while the ASD cohort had a mean of 2.25 bad channels per file (range = 0–4). A 60 Hz notch filter was applied to remove line noise, followed by a 1–120 Hz band-pass filter to attenuate low-frequency drift and highfrequency noise while preserving ERP-relevant activity. This range was chosen as a conservative threshold, as scalp EEG potentials rarely exceed amplitudes of +/− 100 μV as do the associated frequencies of potential interes^55^.

#### Event detection and epoching:

Epochs were created from −500 ms to 1000 ms relative to trial onset. This extended window was intentionally chosen to fully encompass both the participant’s response period and the variable inter-stimulus interval that preceded the next trial, ensuring that as much task-relevant activity as possible was captured in each epoch. Subsequent analysis was restricted to a narrower window, to remove activity that may be related to the response in the preceding trial or to the onset of the subsequent trial. No baseline correction was applied at this stage to preserve raw signal characteristics for subsequent preprocessing.

#### ICA Artifact Removal:

To remove artifacts from eye movement, muscle activity, and other nonneuronal sources, independent component analysis (ICA) was performed using FAST ICA with unlimited components, random state 42, and max 1000 iterations^56–58^. ICA components were visually inspected using the time courses of the components. Artificial components identified by spatial topography consistent with blinks, muscle activity, or channel artifacts were manually selected for exclusion by the experimenter. Across files with ICA metadata available, the NT cohort had an average of 1.50 removed ICA components per dataset (range: 0–4), while the ASD cohort had an average of 1.75 components removed (range: 0–5).

#### Epoch rejection and data retention:

Following ICA correction, automated epoch rejection was performed using Autoreject^59^. Autoreject uses cross-validation to learn channel-specific noise thresholds and automatically repair or reject contaminated epochs to minimize human bias and improve processing quality. The algorithm tested for one, two, or four channel interpolations per epoch and selected the optimal strategy to maximize data retention while maintaining signal quality. Overall, AutoReject rejected on average 9.45% of epochs due to data quality issues (NT: 6.84%, ASD: 12.81%). Because the rejection rate was well below 50% in all experimental blocks, no blocks were excluded due to these flags. Clean epochs were saved in MNE Python (.FIF) files for subsequent analysis, and pre-processing metadata were saved as JSON files for tracking and quality control.

In addition to signal quality, a subset of epochs with unusually long reaction time were excluded from subsequent analysis because they could denote lapses in attention or periods when the participants took a voluntary break without communicating it. Specifically, epochs corresponding to the slowest 10 percent of reaction times were excluded, followed by exclusion of all epochs with reaction times exceeding 2.5 seconds.

#### Event Related Potential (ERP) Generation:

ERP preprocessing was performed separately for each experimental block, i.e. for each participant and image. Neural activity in each epoch and channel was re-referenced to the global average (subtracting the mean of all EEG channels at each time point), then averaged across epochs. Baseline correction was applied to each channel by subtracting the mean amplitude of that channel within a pre-stimulus window extending from −100 ms to 0 ms. Because in some trials the inter-trial interval could be less than 100 ms, the estimate of the baseline activity could be contaminated by activity related to the previous trial’s response. To ensure that this did not affect the data presented in Results, we repeated all analyses using a shorter window of −25ms to 0.0ms which does not include the previous trial response, and obtained similar results.

For the analyses of Section 2.2, we averaged the ERP across selected channels to create a single ERP per image and per participant. In other analyses, we further aggregated data across different participants that segmented the same image, resulting in one ERP per image. Lastly, in other analyses we aggregate across different participants and images, resulting in a single ERP. The aggregation was always conducted separately for NT and for ASD participants.

#### Topographic Maps:

To study the topography of ERPs, topographic maps were generated from individual evoked responses using all available EEG channels. Grand-average topographic maps were computed separately for NT and ASD cohorts using MNE-Python’s grand-average function. We also computed NT - ASD difference maps. All maps were displayed on a consistent −10 to +10 μV color scale, baseline-corrected from −100 to 0 ms, referenced to a global average reference, and filtered using the reaction-time exclusions explained above.

### Gaze data acquisition and preprocessing

#### Eye tracking:

For a subset of participants (16 subjects, for a total of 82 blocks in the NT cohort; and 4 subjects for a total of 26 blocks in the ASD cohort), we monitored gaze using an EyeLink 1000 system (SR Research, Mississauga, ON, Canada) at a sampling frequency of 500Hz. A 9-point calibration was performed at the start of every experimental block. Gaze data were collected throughout the entire block and analyzed separately in different epochs as explained below.

#### Preprocessing:

Gaze data were preprocessed and analyzed using MNE-python. During preprocessing, blinks and gaze data outside of the image area were removed. Sessions were manually checked for quality: those with poor calibration and/or gaze density that appeared unrelated to the image were excluded (18/82 blocks for NT, 13/26 blocks for ASD).

Each experimental block comprises different epochs, for which gaze data was collected and can be analyzed separately. These include: reading the text instructions; initial viewing of the image; trials epochs (between cues onset and response, see Fig. 1). Analysis reported in this paper focuses on gaze density during the initial viewing of the image. Gaze density was calculated from the gaze samples’ spatial coordinates using Gaussian kernel density estimation over the image area with a smoothing standard deviation of 10 pixels. Gaze density maps were then downsampled to the same spatial resolution as the segmentation maps (i.e. 15×15).

### Statistical significance

Different types of statistical significance tests were required for different data modalities and analyses. Therefore, the details are provided in Results.

## RESULTS

### 1. Spatial characteristics

#### 1.1 Perceptual segmentation maps

**Figure 3.**
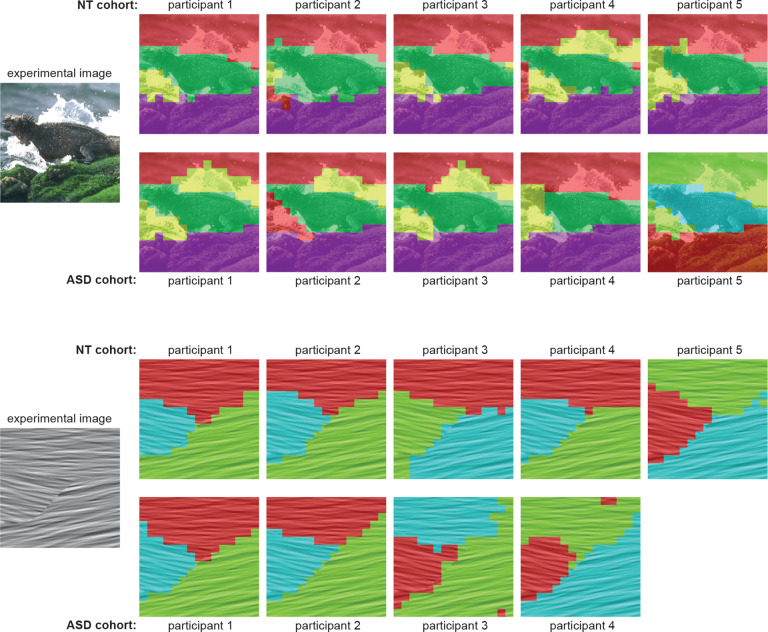
Example segmentation maps. **Top,** a comparison of perceptual segmentation maps for 5 NT participants and 5 ASD participants segmenting the same natural image (shown on the left). The colors corresponding to segment labels are overlaid on the image to facilitate visualization. The numerical values of the labels, and corresponding colors, do not convey ordinal or semantic information and thus should not be compared across participants. **Bottom,** A comparison of segmentation maps for 5 NT participants and 4 ASD participants segmenting the same synthetic texture image (shown on the left).

Our first goal was to determine if participants in both cohorts completed the task successfully. For each participant and image, we used the numerical procedure described in Methods to reconstruct the perceptual segmentation map (Fig 2C) from the response data collected in the corresponding experimental block. Figure 3 demonstrates qualitatively that meaningful maps could be reconstructed for participants in both cohorts. The maps show alignment with meaningful elements of the natural image (e.g. the lizard, rocks, and water in Fig. 3, top) and with boundaries defined by local orientation differences in the texture (Fig. 3, bottom). As a control, we confirmed that no meaningful maps could be reconstructed when participants’ responses were randomly reshuffled across trials to make them unrelated to the visual cues (Supplementary Fig. 1B). Across our dataset, meaningful maps could be reconstructed for both cohorts in the vast majority of the experimental blocks (139/143 blocks for NT; 67/76 for ASD). In the remaining blocks, map reconstructions revealed that the participants responded randomly, i.e. they ignored the task instructions (Supplementary Fig. 1A).

Although the maps were similar between individuals and cohorts, there were also noticeable differences in the details. For instance, in Fig. 3, top, some participants treated the water splash as a single segment on its own, others merged it with the background water, and others yet split the splash into two separate segments due to visual occlusion. To quantify the similarity of the segmentation maps between participants, we defined a measure termed Information Gain (IG; detailed in Methods): intuitively, given two segmentation maps, the IG denotes how well the spatial organization of one map can be predicted from the other. A large percent IG value indicates that two maps are more similar between themselves than to surrogate maps in which pixel locations are shuffled randomly. On the other hand, an IG of 0% means the two maps are not more similar than two random maps.

**Figure 4.**
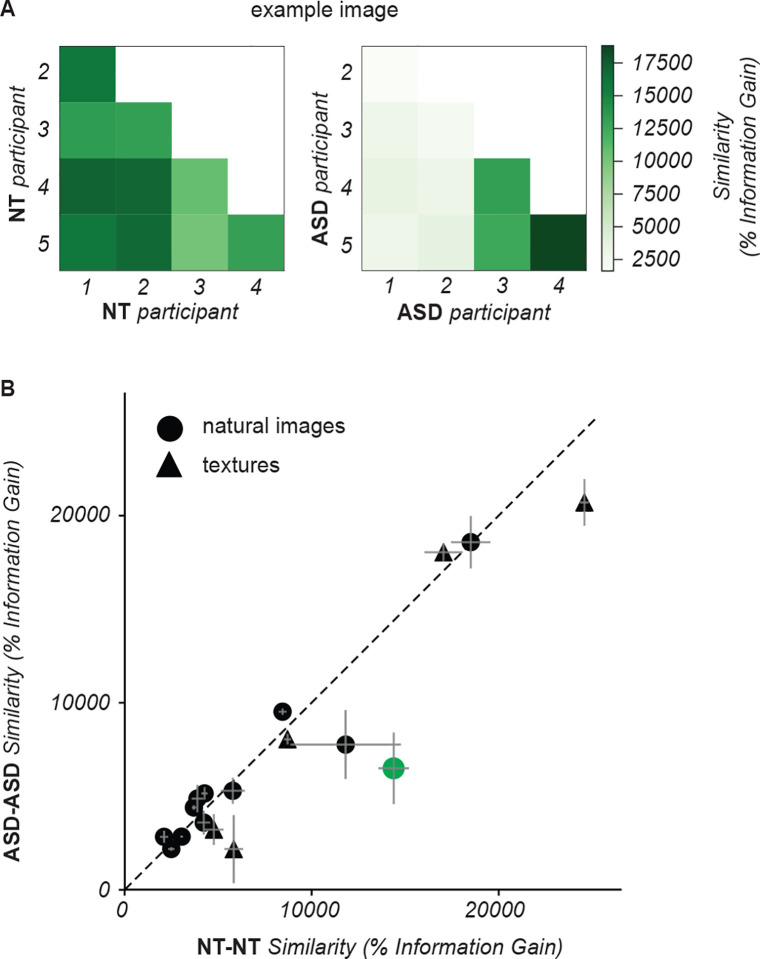
Similarity between the segmentation maps of different participants. **(A)** Each entry in the matrix represents the similarity between the segmentation maps of a single image by two participants in the NT cohort (left) and in the ASD cohort (right). Similarity was measured by information gain (IG), namely how much better the segment labels in one map can be predicted from the other map than from randomly shuffled maps (see methods). Darker shades of green correspond to higher IG, i.e. higher similarity. **(B)** Each symbol represents the mean IG for one image, averaged across all pairs of NT (abscissa) and all ASD (ordinate) participants that segmented that image. The green symbol corresponds to the example image in **(A)**. Error bars indicate the standard error of the mean across pairs of participants per image.

We compared quantitatively between cohorts the inter-participant similarity of the maps using IG. Figure 4A illustrates, for one image, that IG values were often larger between pairs of NT participants than between pairs of ASD participants (mean IG = 14,383 +/−821% s.e.m. between all pairs of maps from 5 NT participants; mean IG = 6,494 +/−1,915% s.e.m. between all pairs of maps from 5 ASD participants). Across the entire dataset, we observed large values of IG in both cohorts, denoting that the maps were consistent between participants (Fig. 4B). This was the case both for natural images and textures. For some images, we observed higher similarity between NT participants than between ASD participants, whereas the opposite effect was never observed. Across all images, the difference between cohorts was not significant (mean IG 7396.30 +/−1452.6 % for ASD; 8450.8 +/−1596.1 % for NT; p=0.628).

In summary, qualitative inspection of the segmentation maps indicates that both cohorts performed the segmentation task successfully and perceived meaningful segments in the images. Quantitatively, there was high similarity in the spatial organization of perceptual segments between individuals. For some images the similarity was lower between ASD participants denoting more idiosyncratic segmentations.

#### 1.2 Perceptual uncertainty and self-consistency

**Figure 5:**
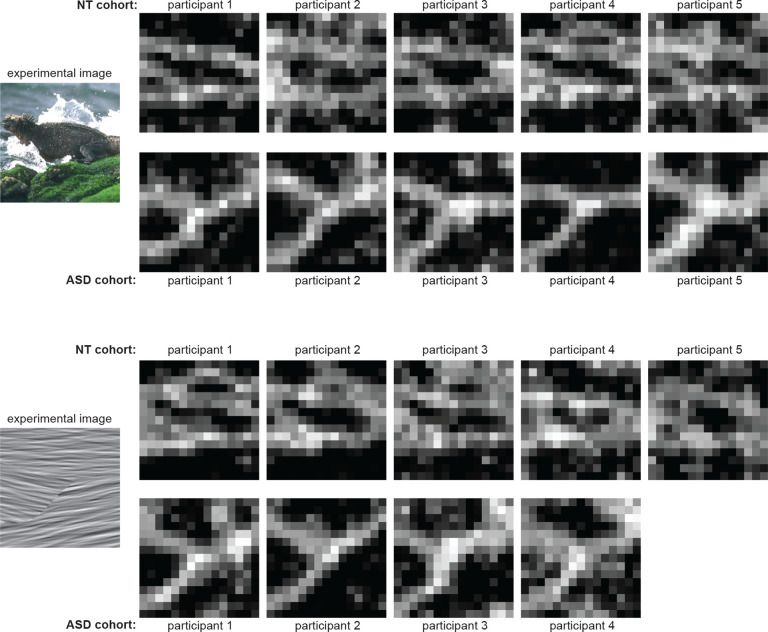
Example uncertainty maps. Uncertainty maps (defined in Fig. 2D) are shown for the same images and participants of Fig. 3.

The segmentation maps analyzed in the previous section represent the most likely subjective interpretation of the image. Our measurements also convey richer information about the perception of segments. First, the reconstruction procedure (detailed in Methods) computes a probabilistic segmentation map (Fig. 2, left) from the trial-by-trial responses: namely, for each pixel we obtain an estimate of the probability that that pixel was assigned to any one of the possible segments. A pixel with a probability of 1 for a given segment, and 0 for all other segments, indicates that the participant was highly certain about that pixel. On the other hand, a pixel with equal probability for multiple segments denotes high uncertainty. We quantified this subjective uncertainty with the entropy of the probability values per pixel (higher values of entropy correspond to higher uncertainty; detailed in Methods). Figure 5 illustrates the segmentation uncertainty maps for the same images and participants as Fig. 3. Just like the segmentation maps, the uncertainty maps were qualitatively consistent between individuals for both the natural image and the texture.

**Figure 6.**
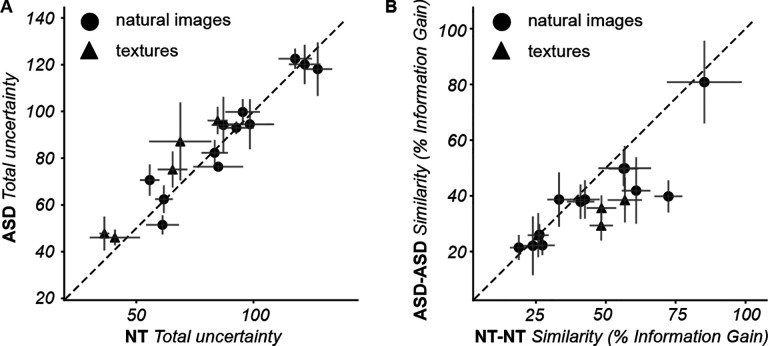
Comparisons of perceptual uncertainty between participants and cohorts. **(A)** Each symbol represents, for one image, the total uncertainty across all pixels in the image averaged across all NT (abscissa) and all ASD (ordinate) participants that segmented that image. Error bars indicate the standard error of the mean across participants per image. **(B)** Each symbol represents the mean IG between uncertainty maps for one image, averaged across all pairs of NT (abscissa) and all ASD (ordinate) participants that segmented that image. Error bars indicate the standard error of the mean across pairs of participants per image.

We compared the overall subjective uncertainty between cohorts. We summarized each uncertainty map into a single value by computing the average across all pixels, which represents the overall subjective uncertainty for that particular image. Fig. 6A shows that for most natural images, the overall uncertainty level was similar between NT and ASD participants (across all 12 natural images: mean uncertainty 90.45 +/−6.6 for ASD; 90.66 +/−6.83 for NT; ttest p=0.982). Conversely, texture images induced systematically larger uncertainty in the ASD cohort, although the difference between cohorts was not significant (across all 5 texture images: mean uncertainty 70.46 +/−10.2 for ASD; 59.25 +/−9.0 for NT; t-test p=0.434).

We then compared the spatial organization of uncertainty, using the same IG metric as for the perceptual segmentation maps. We observed that the spatial organization of uncertainty varied between individuals in each cohort (Fig. 6B), i.e. generally lower values of IG for uncertainty maps than for segmentation maps (cfr. Fig. 4B). In addition, for the majority of images the spatial distribution of uncertainty was more similar between NT participants than between ASD participants (Fig. 6B). This was the case for both natural images and textures, although the difference was not significant for either category (across all 12 natural images: mean IG 39.12 +/−4.8 % for ASD; 45.39 +/−6.1 % for NT; t-test p=0.425; across all 5 texture images: mean IG 32.34 +/−2.88 % for ASD; 44.85 +/−6.6 % for NT; t-test p=0.135).

**Figure 7.**
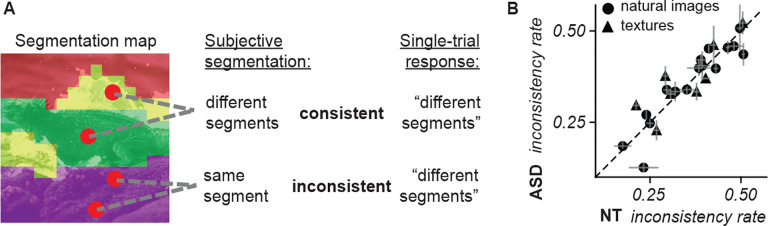
Comparison of self-consistency of responses. **(A)** An example of a consistent response (top pair of red circles) and an inconsistent response (bottom pair of red circles). In both cases, the participant responded “different segments” during the trial, but only one of these responses is consistent with the subjective segmentation map. **(B)** Each symbol represents, for one image, the inconsistency rate (proportion of trials with inconsistent response, over one experimental block) averaged across all NT (abscissa) and all ASD (ordinate) participants that segmented that image. Error bars indicate the standard error of the mean across participants per image.

In addition to the uncertainty maps, we studied the self-consistency of the responses of each participant throughout an experimental block. The segmentation map represents the most likely perceived segments, but single trial responses can be either consistent or inconsistent with the map. For instance, as illustrated in Fig. 7A, in a given trial the response could be “different segment” for a pair of locations that are in the same segment according to the map: this response is inconsistent with the subjective segmentation map. Therefore, by comparing the trial-by-trial responses in an experimental block with the corresponding segmentation map, we classified each trial as consistent or inconsistent and we quantified an inconsistency rate (the proportion of trials with inconsistent responses). The inconsistency rate varied approximately between 0.2 and 0.5 depending on the image, with no systematic differences between cohorts (Fig. 7B). The fact that the majority of responses were consistent indicates that participants performed well at the task. Nonetheless the proportion was relatively high (a rate of 0.35 across all blocks; 21,453 inconsistent trials out of 60,722 for NT, 14,122 out of 39,521 for ASD), likely due to the intrinsic ambiguity of the segmentation problem and the short presentation time, which made the task difficult. Importantly, these inconsistencies did not indicate just response ‘noise’ or randomness (otherwise we would not be able to reconstruct meaningful segmentation maps; see Supp. Fig. 1), but rather subjective uncertainty with a meaningful spatial organization.

In summary, our measurements of uncertainty and response self-consistency provide additional dimensions to characterize the subjective nature of perceptual segmentation. At the coarse level of an entire image and experimental block, the uncertainty and self-consistency were similar between cohorts, further supporting the applicability of this paradigm to the ASD population. Yet, the richer information in the spatial maps revealed that subjective uncertainty is concentrated in similar parts of a given image when comparing NT participants, but this spatial allocation of uncertainty differs substantially more between ASD participants.

#### 1.3 Gaze maps, spatial coverage/spread

Eye movements are frequent perceptual decisions that vary person-to-person, moment-tomoment, and are highly driven by image content/salience. To study how these perceptual decisions may vary between cohorts, we recorded eye-tracking data in a subset of experimental blocks (83 blocks in the NT cohort and 26 blocks in the ASD cohort). Each block begins with an initial viewing period of the image to be segmented (Fig. 1, left); the amount of time spent in this epoch is determined by the participant. On average, ASD participants viewed the image for 10.58 +/−7.2 seconds (st. dev.); NTs viewed for 8.96 +/−5.18 sec.

**Figure 8:**
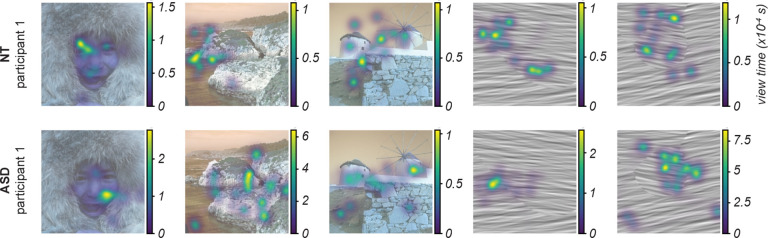
Example gaze density maps. Gaze density is represented by the colored overlay on each image and was computed as the total amount of time that gaze spent on each pixel of the image, during the initial viewing of the image for up to 10 seconds (Fig. 1, left). Each map was calculated using Gaussian kernel density estimation over all image pixels and a smoothing factor (sigma=10 pixels).

To characterize how visual attention was allocated to different parts of each image, we computed gaze density maps which measure view time per pixel. Figure 8 demonstrates example gaze densities during the initial image viewing epochs of two participants (rows) viewing 5 images (3 natural and 2 texture images; columns). We observed qualitative differences for some images. For instance, when viewing the image of a face (Fig. 8 column 1), the NT participant’s gaze was concentrated around the face’s eyes, whereas the ASD participant viewed other face regions. This appears consistent with known differences in ASD viewing behavior in social contexts^40^. Which image regions were the most salient (i.e. highest gaze density) also differed between the two example participants in some non-social natural contexts (e.g. landscape image, Fig. 8 column 2) and texture images (e.g. Fig. 8 column 4) but was qualitatively similar in others (Fig. 8 columns 3,5). These examples illustrate how this paradigm can be used to explore the possibility that differences in ASD visual saliency processes extend beyond social contexts.

**Figure 9:**
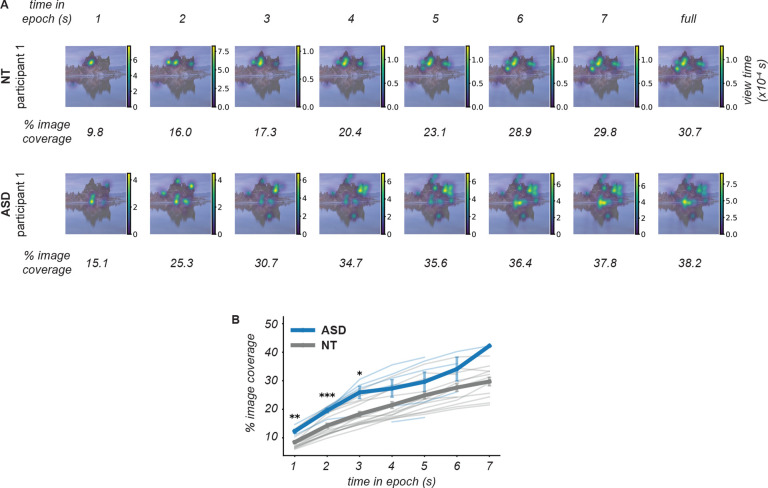
Comparison of image coverage by gaze during initial image viewing. **(A)** Gaze density is represented as in Fig. 8, for one example image. Each panel uses gaze data from a cumulative time window beginning at time 0 and extending for a duration indicated in the top row (time in epoch). The numbers underneath each panel quantify the percentage of the image area that has been visited by the gaze during the corresponding time window. **(B)** Each thin line represents, for one image, the mean across all participants in one cohort that viewed that image. Thick lines represent within-cohort means. Error bars indicate the standard error of the mean across participants and images, within each cohort. Note that total view time was determined freely by each participant, therefore the mean is taken over a smaller number of participants at longer times in epoch. *p<0.05; **p<0.01; ***p<0.001.

To quantify the spatial allocation of visual attention regardless of image content, we measured what fraction of spatial area of each image was viewed by each participant (% image coverage). Across all images, we found that ASD participants tended to view a greater spatial area of the image: Gaze densities during the entire initial viewing epoch on average covered 38.5+/−3.0% of the image for ASD participants (n=26 blocks), vs 31.5 +/−1.4% for NT participants (n=82 blocks) (two-sided t-test p=0.038). We asked if this difference could be explained by differences in viewing epoch duration, or if greater spatial coverage in ASD is also present on smaller time scales. To address this, we calculated gaze density and % image coverage at increasing time periods in the epoch (Fig. 9A) and found that the cohort difference was present as early as the first second of image viewing (Fig 9B). Thus, our results show differences in ASD spatiotemporal gaze dynamics on short, moment-to-moment timescales as well as during longer periods of image viewing.

### 2. Temporal characteristics

#### 2.1 Reaction times

**Figure 10.**
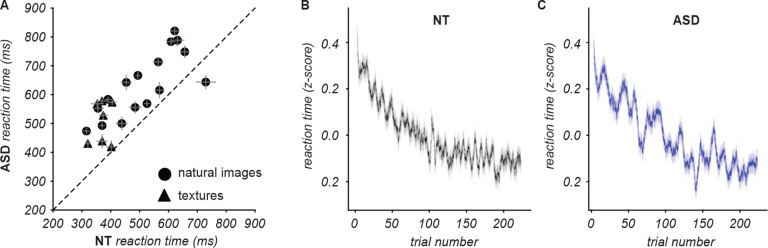
Comparisons of reaction times. **(A)** Each symbol represents, for one image, the mean reaction time averaged across trials in the experimental block and all NT (abscissa) and all ASD (ordinate) participants that segmented that image. Error bars indicate the standard error of the mean across participants per image. **(B)** Reaction time for the first 225 trials across all experimental blocks for NT (left) and ASD (right) participants. Reaction times were z-scored per experimental block before combining across blocks. Trials were ordered by their trial number within each block (i.e. there were many trials indexed “trial 1” across all stimuli). A sliding window of 500 was used across all of these trials. Mean: The solid line and shaded area indicate, respectively, the mean and standard error of the mean within the sliding window.

A common finding in ASD research on perceptual decision making is that ASD participants often respond more slowly than NT. Interestingly, the opposite pattern is sometimes observed in tasks related to segmentation such as the Embedded Figures task^60^, the Wechsler Block Design^17,61^, and visual search of a target among distractors (a form of figure-ground segmentation)^62,63^. In our task, the reaction times were compatible with the more commonly reported effect: Across all trials the median reaction time was 445 ms (95% C.I. of 5 ms) for the NT cohort versus 572 ms (95% C.I. 7ms) for the ASD cohort. This effect was evident for all but one image (Fig. 10A). Although the absolute reaction time values varied by a factor of two or more within each cohort, the difference between cohorts did not appear to scale with the reaction time, i.e. the difference did not increase for images that took longer to segment. Furthermore, trials with inconsistent responses took longer than those with consistent responses, but there was no significant difference between cohorts: inconsistent trials were 53 ms (95% CI: 8ms) longer in NT, and 45 ms (95% CI: 12 ms) longer in ASD.

One potential concern when comparing the reaction times between cohorts is that ASD participants could have greater difficulty maintaining focus on the task throughout the entire duration of a block. If that was the case, their reaction times should increase as the block progresses, or oscillate widely as attention wanes and wades. To rule this out, we compared between cohorts how reaction times changed over trials throughout the block. In both cohorts, reaction times decreased monotonically with trial number (Fig. 10B). This finding indicates that there was no decline in focus or engagement throughout the block; on the contrary, participants rapidly improved their performance. To compare this effect quantitatively, we fit an exponential function and computed a “trial constant” enumerating, in each cohort, how many trials participants required to halve their reaction time (details in Methods). The trial constant for the NT cohort was significantly shorter, namely 18.4 trials (95% CI 0.4) versus 21.9 trials (95% CI 0.7) for the ASD cohort, suggesting NT participants learned the sensorimotor contingencies slightly faster. Note that an entire block lasted between 225 and 900 trials, therefore the difference between cohorts was less than 1% of the total number of trials in the block.

In summary, our measurements of reaction times reveal a systematic and sizable difference in the dynamics of perceptual segmentation between cohorts. This difference cannot be attributed to a disengagement from the task or deficit of attention in the ASD cohort, indicating that our task reveals meaningful differences in perceptual processing and decision making.

#### 2.2 Neural dynamics in visual areas

In a subset of participants (n = 11 NT, 9 ASD) we recorded EEG data while they were performing the task. Because segmentation is known to involve recurrent interactions within occipital and parieto-occipital regions^17,64,65^, we examined activity from occipital and parietooccipital electrodes (O1, Oz, O2, PO7, PO3, POz, PO4, PO8). We focused on event-related potentials (ERPs) in those electrodes by aligning activity to trial onset. Details of ERP calculation are provided in Methods.

**Figure 11.**
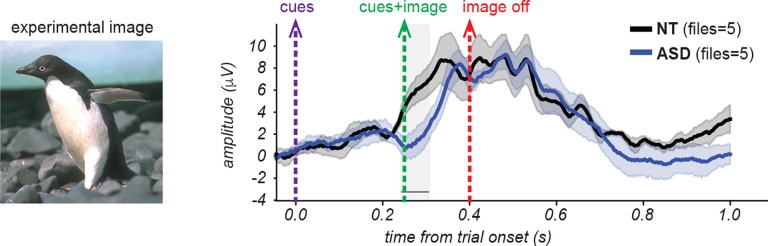
Comparison of event related potentials for one example image. ERPs from occipital and parieto-occipital electrode sites (O1, Oz, O2, PO7, PO3, POz, PO4, PO8) for the example image on the left, comparing NT (n=5 files; black) and ASD (n=5 files; blue) cohorts. Waveforms are time-locked to the beginning of the trial (blue vertical line). Lines represent the ERPs averaged across participants, within each cohort. Shaded regions around the ERPs indicate standard error of the mean. The gray vertical band highlights a significant cohort difference (240–326 ms; p < 0.05, point-by-point independent samples t-tests).

**Figure 12.**
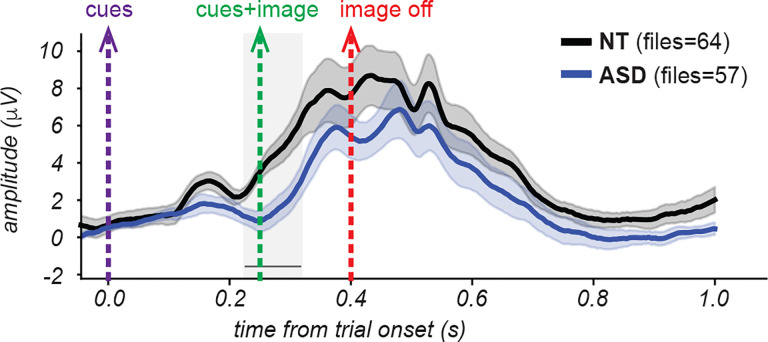
Comparison of grand-average event related potentials for one example image. Grandaverage ERPs from occipital and parieto-occipital electrode sites (O1, Oz, O2, PO7, PO3, POz, PO4, PO8) across all images and participants, within each cohort. Same conventions as in Fig. 11.

Figure 11 illustrates the ERP waveforms for both cohorts (5 NT and 6 ASD participants) in response to a single image for which participants were to divide into 4 segments. Both cohorts exhibited a prominent positive-going deflection peaking between approximately 300–400 ms after the onset of the trial. Recall that at the start of the trial the two cues are presented on a gray screen for 250 ms, followed by 150 ms with the same two cues now superimposed on the image. Compared to the ASD cohort, the NT cohort showed an earlier onset of the voltage deflection and higher amplitude responses throughout the time course. These effects were evident also for the grand average ERP across all stimulus conditions (Fig. 12; n = 11 NT subjects for a total of 64 blocks, 8 ASD subjects for a total of 57 blocks) as well as for the majority of other unique images (Supplementary Fig. 2).

To statistically evaluate these differences, we conducted point-by-point independent samples t-tests comparing NT and ASD cohorts at each time point using subject level ERP waveforms. A significance threshold of p < 0.05 was used to identify time windows of cohort differences, with a minimum duration criteria of 50 ms to reduce the influence of transient fluctuations.

For the specific image ERPs shown in Fig. 11, this analysis revealed significant cohort differences in the time window spanning 240.2–326.2 ms. When examining the grand average across all images at O/PO electrodes in Fig. 12, significant differences were found in a similar time window (222.7–324.2 ms).

In summary, these findings indicate that in our dataset the NT cohort robustly demonstrates faster onset and enhanced amplitude ERPs in O/PO channels relative to the ASD cohort during a temporal window that immediately precedes and includes the presentation of the image.

#### 2.3 Brain-wide neural dynamics

Based on prior work illustrating that individuals with ASD exhibit atypical fronto-occipital connectivity in fMRI^17,66–68^, and that these connectivity patterns correlate with segmentation performance^17^, we focused our second set of analyses on the full spatiotemporal neural dynamics across the scalp. This allowed us to test whether significant cohort differences emerged in the spatial organization of activity across all channels and at other specific channels besides the O/PO cluster.

**Figure 13.**
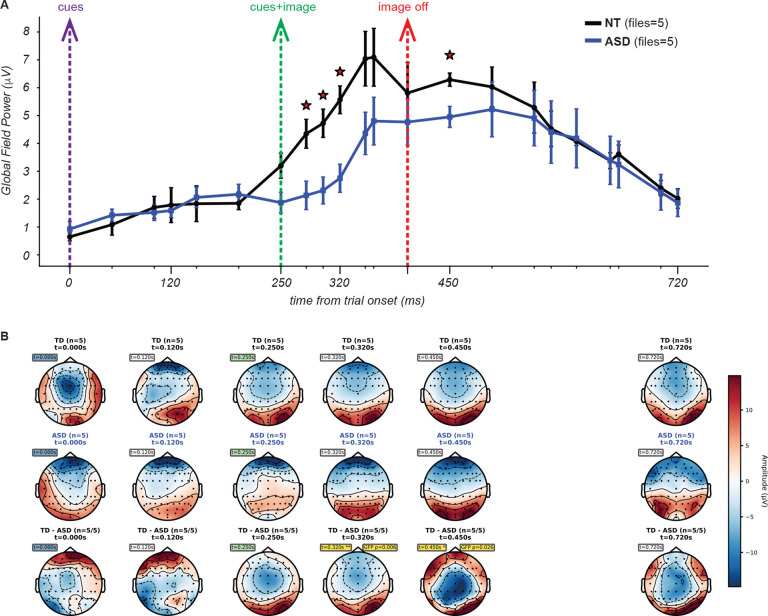
Comparison of Global Field Power and Topographical Maps for one example image. **(A)** Global Field Power (GFP) for the same image as Fig. 11. GFP was calculated as the spatial standard deviation of the electric field across all EEG electrodes at each time point, using the formula: √[mean((amplitude - mean(amplitude))^2^)] across all electrodes. Black and blue lines represent the mean across participants within each cohort, error bars indicate the standard error of the mean. Vertical dotted lines indicate experimental events, as in Fig. 11. Red stars indicate time points with statistically significant GFP differences between cohorts (p < 0.05, independent samples t-tests). **(B)** Topographical maps showing scalp distribution of electrical potential at selected time points corresponding to experimental time points and points where GFP was statistically significantly different between cohorts. Three rows display: NT cohort topomaps (top row), ASD cohort topomaps (middle row), and difference topomaps (NT - ASD, bottom row). Solar scale ranges from −10 to +10 μV, with red indicating positive amplitude and blue indicating negative amplitude. Red stars on GFP plots mark timepoints with significant pointwise GFP t-test differences (p < 0.05, uncorrected). Time point labels on the difference topomaps are similarly colored to match experimental events (blue for 0ms, green for 250ms, red for 400ms), with gold boxes and asterisks (**,*) indicating GFP significance levels (p < 0.01, p < 0.05 respectively). GFP p-values are displayed in gold boxes next to the time point labels for topomaps with significant differences.

**Figure 14.**
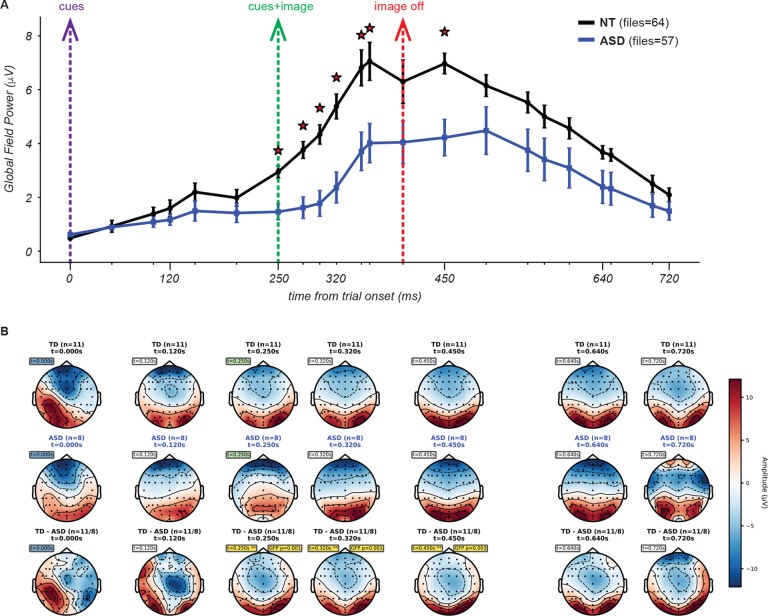
Comparison of Global Field Power and Topographical Maps. GFP and topographical maps comparing NT (n=11 participants) and ASD (n=9 participants) cohorts, averaged across all stimulus conditions. Data processing and display conventions are identical to Fig. 13.

To statistically quantify cohort differences in the spatial pattern of response amplitude across the scalp, we computed Global Field Power (GFP) at 50ms intervals throughout each time window. GFP reflects the spatial standard deviation of the scalp electric field at each time point and provides a channel-agnostic index of the overall strength and spatial differentiation of the neural responses^62^. GFP values were computed for each participant at each time point, and NT vs ASD differences were assessed using independent samples t-tests (p < 0.05). This analysis identified a consistent window of significant cohort differences from roughly the onset to the offset of the image presentation (i.e. 250 ms to 400 ms) as well as roughly 50 ms after image offset, both for the example single-image condition (Fig. 13A) and the grand average (Fig. 14A). In addition, NT participants exhibited earlier onset of the rising phase of GFP, even preceding image onset (Fig. 14A), and overall higher values of GFP than ASD participants. These observations further corroborate the ERPs findings, given that the times of significant differences occur well before responses were reported (average reaction times were several hundreds of ms after image offset, as shown in Fig. 10). Therefore, activity in this time window is expected to reflect primarily task expectations, visual processing and evaluation but not reflect planning and execution of the delayed motor response (key press).

To descriptively characterize where the two cohorts differed in the spatial pattern of neural responses, we generated topographical maps of activity (topomaps; details in Methods) for each cohort, as well as difference topomaps between cohorts. These maps provide a scalplevel view of the data and complement the ERP waveforms and GFP by showing where activity differs, not just when. We visualized these differences using topomaps at key points identified by the GFP analysis (Fig. 13B, 14B). Time points were chosen based on (1) major experimental events, (2) GFP-significant time points, and (3) additional points close to image onset to improve temporal resolution around the early visual processing period. The maps for the example condition (Fig. 13B) reveal a spatially coherent posterior difference present at the times of significant GFP differences. The grand-average topographic maps demonstrated this pattern even more robustly (Fig. 14B). Lastly, for a quantitative analysis of differences in the spatial distribution of activity, we performed point-by-point electrode level t-tests and cluster based permutation tests. Many individual electrodes showed significant pointwise effects (p < 0.05) across an extensive swath of posterior occipital electrodes (Supplementary Table 1).

This spatial analysis revealed that differences localized to the occipital and posterior occipital channels. However, past work on perceptual segmentation with fMRI identified activity differences also in brain regions beyond visual cortex^17,64^. We reasoned that differences in channels other than O/PO should be more prominent in later time windows than the one considered above. Furthermore, differences in feedback-driven and predictive mechanisms may unfold well after the time window considered here^14,66^. Indeed, the grand-average GFP indicated higher power in the NT than ASD cohort at all times in the later part of the trial, but this difference did not reach statistical significance. The topomaps at the latest time point analyzed (720 ms) indicate also a noticeable qualitative difference in frontal channels, although not significant (see Discussion for further interpretation).

Together, these results show a clear and spatially cohesive pattern of cohort differences in posterior occipital and parieto-occipital regions during visual processing. The timing aligns with the significant ERP difference window observed in the ~220–330 ms window. The topomaps visually illustrate that the effect spans a broad posterior region rather than being confined to a small region. The convergence of the ERPs, GFP, and pointwise electrode statistics strengthens the reliability of this effect and indicates a consistent alteration in the spatial profile of visual processing in ASD during natural scene and texture segmentation.

## DISCUSSION

We have presented a novel paradigm to study perceptual segmentation of natural images in autism. Segmentation of visual inputs is a fundamental operation of the visual system, enabling observers to organize complex scenes into meaningful parts. Despite its importance, this process has traditionally been studied using simplified, synthetic stimuli, which limit our ability to understand how segmentation unfolds in realistic environments. Natural stimuli are not only a more representative model of everyday visual experiences, but there is also strong evidence that the visual cortex has adapted to the statistics of rich and ambiguous natural scenery^70,71^. Studying natural stimuli therefore allows us to examine, with greater depth, the role of perceptual and neural dynamics within behavioral processes.

We have demonstrated that participants in both NT and ASD cohorts understand and complete the task as designed. Qualitatively, our analysis of gaze and neural data further demonstrate that the perceptual task has been successfully integrated with simultaneous eye tracking and EEG recordings. Our characterization analysis reveals interpretable cohort-level differences between NT and ASD participants, including slower (Fig. 10) and more idiosyncratic (Figs. 4,6) perception in ASD; as well as altered spatiotemporal gaze (Figs. 8,9) and neural (Fig. 11–14) dynamics. By combining highly controlled, trial-based psychophysical measurements with complex stimuli and multimodal data, the paradigm presented here can therefore be applied both for exploratory and hypothesis-driven studies of perceptual organization in ASD.

Our paradigm builds on a design used in many prior studies of perceptual segmentation, namely the “same segment / different segment” task^71,72^. Different from those past studies and other work on segmentation in autism, the paradigm we adopted enables measuring the spatial organization of perceptual segments and their associated uncertainty, through estimation of the segmentation maps (Fig. 2). One clear demonstration of the importance of obtaining these new measurements, is our finding that although perceptual uncertainty (Fig. 6A) and variability (Fig. 7B) appear similar between NT and ASD cohorts, the corresponding spatial maps vary across participants and reveal more idiosyncratic patterns of uncertainty in ASD participants (Fig. 6B). Given our parallel finding that ASD participants explore the images with more spatially distributed eye movements (Fig. 9), it will also be important to study how the spatial organization of segments, uncertainty, and gaze related to each other and to neural variability^73^.

A robust finding between cohorts was that ASD participants took longer to respond to stimuli than NT participants. This finding is consistent with past studies on decision-making in autism^4,17,60^ but our measurements enabled a finer characterization which escapes explanations that rely simply on a generalized slowing down. In particular, we identified a substantial minority of trials in which participants’ responses were inconsistent with their perceptual segmentation maps (Fig. 7). Inconsistent responses took longer in both cohorts, but this inconsistency delay did not differ between cohorts. This would be difficult to explain under theories that assume only a global decision-making delay in ASD^74^, which would predict that inconsistent responses are delayed relative to consistent responses more in ASD than in NT participants. An interpretation is that some subprocesses in segmentation take longer in ASD than NT while others may have more similar timescales.

Preliminary analysis of the neural dynamics to the cue-image sequence revealed a robust response in both groups that was dominant over posterior scalp regions, consistent with generators in visual cortices, and onset at about 225ms and 300ms post-cue, respectively, for the NT and ASD groups. Thus while both NT and ASD participants showed a prominent deflection from 350–450ms after the cue, the NT participants had a substantially earlier response onset (Figs. 11,12). Although one interpretation might suggest more efficient early visual processing in NT participants, this explanation is not fully consistent with larger-sample EEG studies reporting that early visual responses in ASD are often either indistinguishable from NT individuals or differ only subtly^75–77^.

An alternative interpretation supported by recent work is that these early NT-ASD differences reflect top-down predictive or integrative processes rather than differences in basic sensory encoding. Specifically, the fact that NT participants showed earlier activity even before image onset aligns with proposals that ASD participants may rely less on predictive mechanisms or prior expectations when organizing visual information^20,69,78^. Under this view, the early NT ‘advantage’ may reflect anticipatory or expectation-driven processes that facilitate rapid segmentation, whereas ASD participants may require additional time or engage alternate networks in the absence of strong top-down priors. This interpretation is also compatible with the small but noticeable amount of pre-stimulus (i.e. after cues onset but before image onset) activity observed in the ERP (Figs. 11,12) which may arise from anticipatory processes engaged by the presentation of the cues before the presentation of the image.

Importantly, the presentation of the cues is likely to induce a saccade, at least on some trials, e.g. to position gaze in a location that favors solving the segmentation task for the upcoming image. The earlier onset of occipital activity observed in NT participants might partly reflect saccade-related activity during the cue period in NT but not ASD participants. The concurrent measurements of gaze will allow testing in future work if saccades in the cue period are strategically related to the task and if they contribute to differences in neural latencies (i.e. by analyzing fixation related potential^79^). Furthermore, to determine how much of the observed early difference reflects prediction-related mechanisms versus purely evoked visual responses, it will be important to incorporate control conditions with a task in which cue locations are not task relevant (e.g., a color-change detection task at fixation).

Our analysis of ERPs focused on occipital and parieto-occipital channels, because they capture mainly activity in regions canonically involved in early visual encoding, contour integration, and visually driven recurrence^64,80^. This choice was further supported by our topographical results: across analyses, the most reliable cohort differences emerged in O/PO channels (Supplementary Table 1), indicating that these are the spatial locations where cohort-level effects are expressed in this task at this level of analysis. Nonetheless, based on prior studies of segmentation in ASD^17,81^, we also expect differences in a complex neural network that includes higher order cortical regions and in the interaction between brain regions. It is possible that those differences were not apparent in our analysis because we aligned activity to trial onset: the variability of response time across trials could hide effects that are time-locked to the responses.

Our paradigm provides a significant methodological bridge between highly controlled stimuli (illustrated here with the texture-based stimuli) and naturalistic image perception. Previous segmentation work has relied almost exclusively on textures or simplified stimuil^4,15–17,69,81–84^. To our knowledge, this is the first study to examine visual segmentation using both texture based and natural images within the same experimental framework. This distinction is critical to test whether cohort differences in segmentation ability generalize across stimulus types and whether the underlying neural mechanisms differ when the perceptual demands increase. This framework also makes it possible to incorporate images with and without human or animal faces, and with interactions between people, allowing tests of whether social content distinctly modulates segmentation processes, a question motivated by literature on altered social and face-processing mechanisms in ASD^41,81^.

Importantly, our approach is not limited to natural scenes or basic textures. It can also be applied to classical segmentation stimuli such as Wechler block figures^17,85^, coarse face/house images^81^, or Kanisza figures^15^. A major strength of our paradigm is that it generates full segmentation maps rather than binary global judgments. This capability is critical because these stimuli have been central to decades of work on perceptual grouping and illusory contours, yet prior studies have been limited to inferring perception from categorical same/different or present/not present stimuli^80,86–91^. For example, using our method, theories proposing that individuals with ASD perceive the Kanizsa triangle or other illusory contours differently can be tested directly: in addition to inferring perception from binary behavioral judgements, we can now also measure the full segmentation map and its associated uncertainty along perceptual boundaries. Applying our method to these canonical stimuli provides a direct bridge between insights gained from highly controlled artificial stimuli and the more ecologically valid perceptual processes measured in naturalistic scenes. It also creates a unified framework for testing whether the same segmentation mechanisms operate across stimulus classes, or whether cohort differences depend on the specific visual information available.

Our ASD sample consisted of primarily high-functioning individuals. It remains unclear whether similar neural or behavioral patterns would be observed in individuals with differing levels of symptom severity, or, if they would be able to understand and complete the task in its current form. Once a basic characterization of these processes is established in the cohorts considered here, an important next step will be to adapt the paradigm for a broader range of participants, including younger children and individuals with severe intellectual disabilities. Making the task more developmentally accessible through shorter blocks, simplified instructions, or reducing motor demands, will allow us to test whether the same segmentation mechanisms generalize across ability levels and developmental stages. Such adaptations would also create opportunities for longitudinal studies that track the development of perceptual segmentation within the same participants over time.

## Supplementary Material

1
