# Supplementary material for: A Multimodal Framework for Understanding Perceptual Segmentation of Natural Scenes In Autism
Source: bioRxiv. 2025 Dec 18:2025.12.17.695033. Preprint. [Version 1] doi: 10.64898/2025.12.17.695033 (PMC12724402; doi:10.64898/2025.12.17.695033)
Supplement: 1 [file NIHPP2025.12.17.695033V1-supplement-1.pdf]

bioRxiv preprint doi: https://doi.org/10.64898/2025.12.17.695033; this version posted December 18, 2025. The copyright holder for this preprint (which was not certified by peer review) is the author/funder, who has granted bioRxiv a license to display the preprint in perpetuity. It is made available under aCC-BY 4.0 International license.

SUPPLEMENTARY INFORMATION

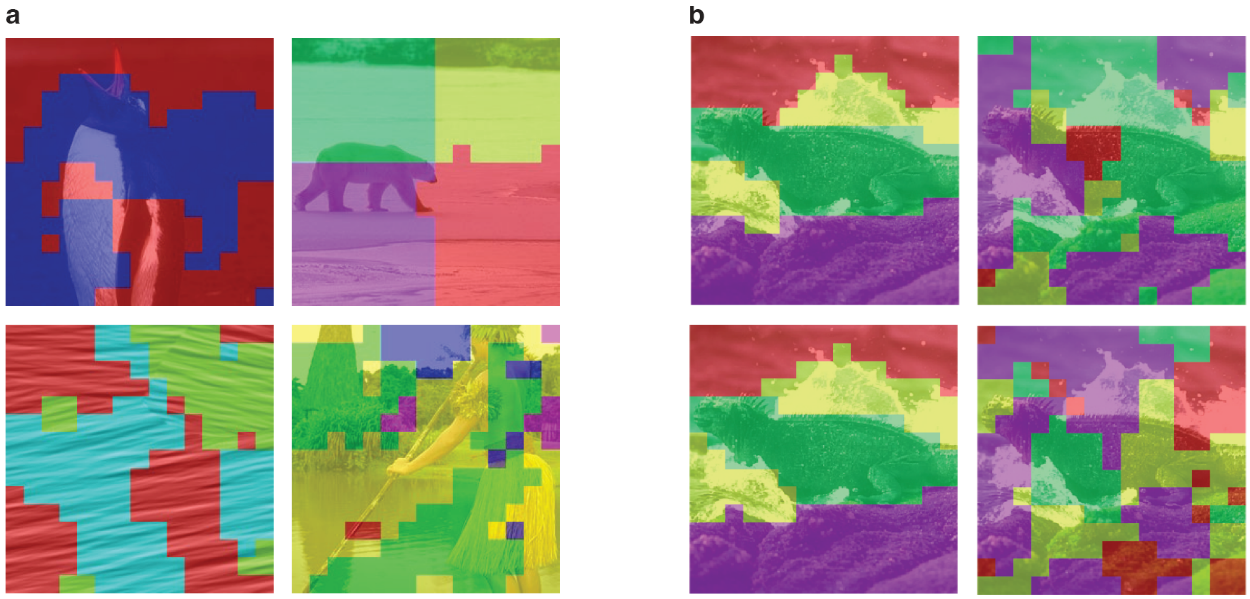

Supplementary Figure 1. (**a**) Examples of maps from blocks excluded from the analysis. We debriefed participants after each block and asked them to indicate verbally the segments they perceived in the image. By comparing those descriptions with the maps we reconstructed numerically, we identified experimental blocks in which the participants did not perform the same-different task: in those cases, the maps do not correspond to the description nor to any meaningful segments. In addition, in one block (top right) the instructions were misinterpreted: on debriefing the participant explained that, because there was no right or wrong answer, they decided to segment the image in four quadrants rather than based on how they perceived it. This is evident from the reconstruction. (**b**) For two participants (rows), examples of maps from blocks included in the analysis (left) and as a control the maps reconstructed after randomly shuffling the order of the participants' responses across trials (right). Trial shuffling destroys the relation between the spatial cues in a trial and the corresponding response from the participant. The resulting maps lack spatial structure and appear unrelated to the image content or to the verbal description. This control analysis supports that the reconstructed maps from the original data are meaningful and not artifactual.

bioRxiv preprint doi: https://doi.org/10.64898/2025.12.17.695033; this version posted December 18, 2025. The copyright holder for this preprint (which was not certified by peer review) is the author/funder, who has granted bioRxiv a license to display the preprint in perpetuity. It is made available under aCC-BY 4.0 International license.

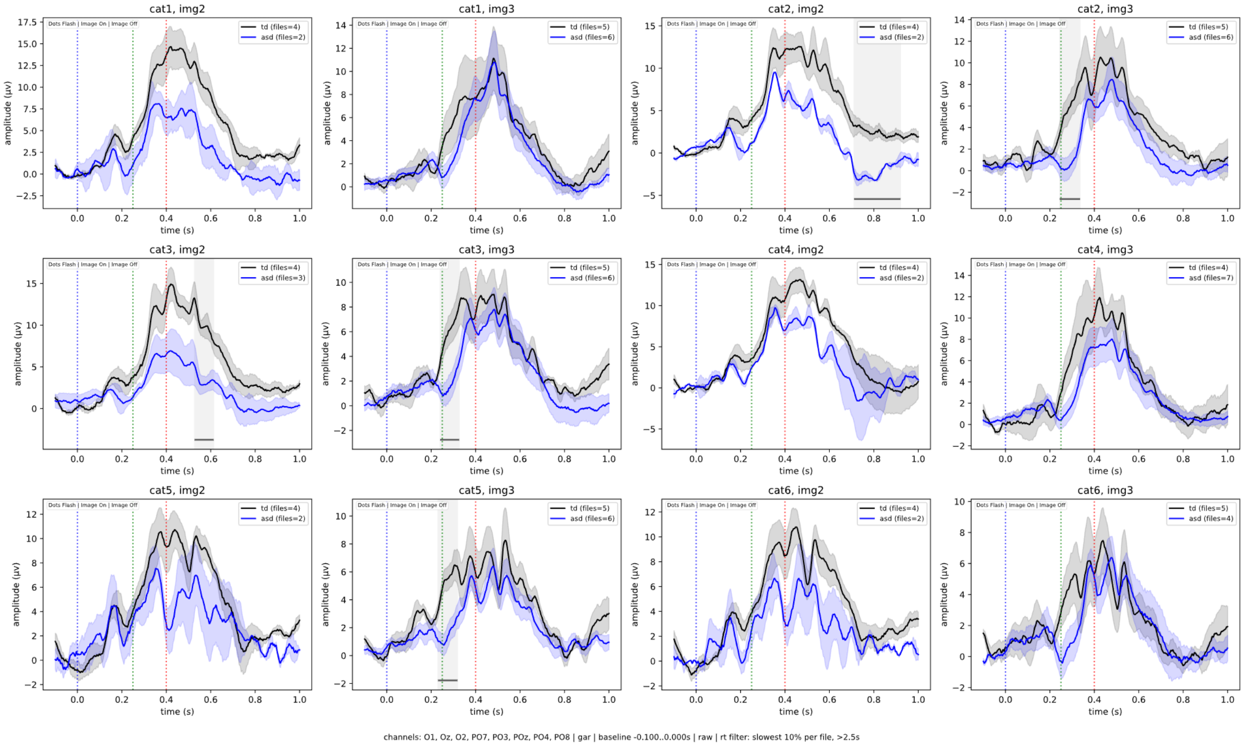

Supplementary Figure 2. Event-related potentials (ERPs) comparing Typically Developing(NT) (black) and ASD (Blue) groups across all experimental images. Grand average waveforms for occipital and parieto-occipital channels (O1, O2, Oz, PO7, PO3, POz, PO4, PO8) are shown for each category-image combination (Categories 1-6, Images 2-3). Black bars at the bottom indicate time windows with significant group differences ($p < 0.05$, uncorrected). Vertical dashed lines mark experimental events: Dots Flash (0.0s, blue), Image On (0.25s, green), and Image Off (0.4s, red).

bioRxiv preprint doi: https://doi.org/10.64898/2025.12.17.695033; this version posted December 18, 2025. The copyright holder for this preprint (which was not certified by peer review) is the author/funder, who has granted bioRxiv a license to display the preprint in perpetuity. It is made available under aCC-BY 4.0 International license.

Grand Average						
GFP p-value	Significance	ROI p-value	ROI Effect Size (d)	N Significant Electrodes	ROI Electrodes (p-values)	Top 5 Other Electrodes (p-values)
0.001347848	**	0.001374147	2.073323491	29	PO8 (0.0005), O2 (0.0005), Oz (0.0022), O1 (0.0024), PO4 (0.0025), PO7 (0.0130)	TP8 (0.0001), FC3 (0.0001), P10 (0.0001), Iz (0.0001), P8 (0.0002)
0.000927539	***	0.002253886	1.953481221	36	PO8 (0.0004), O2 (0.0012), PO4 (0.0029), O1 (0.0055), Oz (0.0058), PO7 (0.0090), POz (0.0489)	P10 (0.0000), P8 (0.0002), TP8 (0.0002), FC3 (0.0003), Iz (0.0003)
0.001190091	**	0.007212254	1.661282998	36	PO8 (0.0012), O2 (0.0075), PO7 (0.0079), PO4 (0.0089), O1 (0.0113)	P10 (0.0001), P8 (0.0008), Iz (0.0008), TP8 (0.0010), FC3 (0.0012)
0.010600359	*	0.078357955	1.008740636	16	PO8 (0.0040), PO7 (0.0328)	P10 (0.0010), TP8 (0.0021), P8 (0.0023), P6 (0.0073), FC1 (0.0086)
0.002969443	**	0.006106457	1.689522889	22	PO8 (0.0002), O2 (0.0006), Oz (0.0058), O1 (0.0107), PO4 (0.0242), PO7 (0.0266)	P10 (0.0000), TP8 (0.0003), P8 (0.0004), Iz (0.0005), FC1 (0.0014)

Single Image (Cat3/Img3)						
GFP p-value	Significance	ROI p-value	ROI Effect Size (d)	N Significant Electrodes	ROI Electrodes (p-values)	Top 5 Other Electrodes (p-values)
0.020045715	*	0.016229924	2.158762807	18	PO8 (0.0045), PO4 (0.0054), O2 (0.0089), Oz (0.0243), O1 (0.0310), POz (0.0324)	P6 (0.0009), P10 (0.0011), P8 (0.0011), TP8 (0.0012), P4 (0.0019)
0.00590006	**	0.016391845	2.111154807	20	PO8 (0.0031), PO4 (0.0046), O2 (0.0083), POz (0.0345), Oz (0.0360), O1 (0.0465)	P10 (0.0002), P8 (0.0003), P6 (0.0003), P4 (0.0015), TP8 (0.0018)
0.026273168	*	0.053596504	1.572244959	11	PO8 (0.0115), O2 (0.0116), Oz (0.0160)	Iz (0.0218), FC3 (0.0251), C2 (0.0261), P8 (0.0280), P10 (0.0290)

Supplementary Table 1. Statistical comparison of NT vs ASD groups across time windows: Global Field Power (GFP), Region of Interest (ROI), and pointwise electrode analyses for Grand Average and Category 3/Image 3 conditions.
